# Pregnenolone 16-Alpha Carbonitrile, an Agonist of Rodent Pregnane X Receptor, Regulates Testosterone Biosynthesis in Rodent Leydig Cells

**DOI:** 10.3390/jox14030071

**Published:** 2024-09-16

**Authors:** Julia M. Salamat, Elizabeth M. Ayala, Chen-Che J. Huang, Frank S. Wilbanks, Rachel C. Knight, Benson T. Akingbemi, Satyanarayana R. Pondugula

**Affiliations:** 109 Greene Hall, Department of Anatomy, Physiology and Pharmacology, College of Veterinary Medicine, Auburn University, Auburn, AL 36849, USA; julia.salamat@bcm.edu (J.M.S.); emortiz1093@gmail.com (E.M.A.); jeff.huang@auburn.edu (C.-C.J.H.); fsw0007@auburn.edu (F.S.W.); rcknight@uab.edu (R.C.K.)

**Keywords:** Leydig cells, MA-10 cells, pregnenolone 16-alpha carbonitrile (PCN), pregnane X receptor (PXR), testis, testosterone, xenobiotics

## Abstract

Leydig cells (LCs) in the testes produce the male sex hormone testosterone (T). Several xenobiotics, including clinical drugs, supplements, and environmental chemicals, are known to disrupt T homeostasis. Notably, some of these xenobiotics are known to activate the pregnane X receptor (PXR), a ligand-dependent nuclear receptor. However, it is currently unknown whether PXR is expressed in LCs and whether PXR activation alters T synthesis in rodent LCs. Therefore, in this study, we sought to determine whether PXR is expressed in rodent LCs and whether pregnenolone 16-alpha carbonitrile (PCN), the prototype agonist of rodent PXR, regulates T biosynthesis in rodent LCs. Hormonal as well as protein and gene expression analyses were conducted in rat primary LCs and MA-10 mouse Leydig cells. Results showed that PXR was expressed at the mRNA and protein level in both rat primary LCs and MA-10 cells. Incubation of rat primary LCs with PCN resulted in a significant decrease in T secretion. This PCN-induced decrease in T secretion was associated with decreased protein expression of key steroidogenic enzymes such as 3β-HSD and CYP17A1. RNA-seq results from MA-10 cells showed that PCN down-regulated the transcripts of steroidogenic enzymes and proteins involved in the T synthesis pathway. Together, these results suggest that PCN, an agonist of rodent PXR, can regulate T biosynthesis in rodent LCs by down-regulating the expression of the steroidogenic enzymes involved in T biosynthesis. Our results are significant as they provide a potential novel mechanism for disruption of testosterone homeostasis by a variety of xenobiotics.

## 1. Introduction

Leydig cells (LCs) comprise only 3–5% of the testis. However, they are the predominant source of testosterone (T), which is important for the development of the male reproductive tract and male secondary sex characteristics. T also plays an important role in many other physiological processes including mood, cognition, and energy output [1]. Therefore, it is critical that proper levels of T are maintained for normal development and physiology.

Many clinical drugs such as rifampicin, tamoxifen, cimetidine, phenobarbital, and dexamethasone have been shown to alter T levels in humans or rodents [2,3,4,5,6,7]. Similarly, supplements, including St. John’s Wort, licorice, ginkgo biloba extract, and genistein, also affect T homeostasis in humans or rodents [8,9,10,11,12,13]. Furthermore, environmental chemicals, such as polychlorinated biphenyl (PCB), bisphenol A (BPA), ethinylestradiol, perfluorooctane sulfonate (PFOS)/perfluorooctanoic acid (PFOA), triclosan, cypermethrin, and methoxychlor, are known to alter T levels in rodents [14,15,16,17,18,19,20,21,22]. In addition to altering T homeostasis, all of the aforementioned xenobiotics have been shown to directly or indirectly activate the pregnane X receptor (PXR). However, it is currently unknown whether PXR is expressed in LCs, which produce the vast majority of T, and whether PXR activation results in altered T synthesis in the rodent male gonad.

PXR is a ligand-dependent orphan nuclear receptor and transcription factor with a promiscuous ligand-binding domain that allows for a wide variety of endobiotics and xenobiotics to bind to and affect its activity [23,24]. PXR primarily contributes to xenobiotic metabolism by regulating drug-metabolizing enzymes and drug transporters [25]. PXR also plays an integral role in the metabolism of endobiotics, such as bile acids, glucose, and lipids, by regulating the expression of the relevant genes involved in these pathways [26,27,28,29].

Notably, PXR activation was previously shown to disrupt both glucocorticoid and mineralocorticoid hormone homeostasis in mice [30]. A PXR-mediated increase in corticosterone and aldosterone levels was associated with an up-regulation of the adrenal steroidogenic enzymes CYP11a1, CYP11b1, CYP11b2, and 3β-HSD [30]. Similarly, PXR activation resulted in reduced circulating levels of androgens via up-regulation of metabolic enzymes SULT2A1 and CYP3As in the prostate gland [31]. We hypothesized that activation of PXR in rodent LCs can lead to an altered T biosynthesis. Thus, we sought to determine whether pregnenolone 16α-carbonitrile (PCN), the prototype rodent PXR agonist, regulates T synthesis in rodent LCs by altering the expression of critical steroidogenic enzymes. Indeed, our results show that PCN regulates T synthesis in rodent LCs by altering the expression of key steroidogenic enzymes.

## 2. Materials and Methods

**Chemicals and plasmids:** Dimethyl sulfoxide (DMSO; ≥99.9%) and Pregnenolone 16-alpha carbonitrile (PCN; ≥97%) were purchased from Sigma–Aldrich. The pcDNA3, FLAG-pcDNA3, pcDNA3-hPXR, FLAG-pcDNA3-hPXR, and 3XFLAG-pcDNA3-hPXR plasmids were previously described [32,33,34].

**Isolation and culture of rat primary Leydig cells:** Male Long-Evans rats were obtained from Harlan Teklad (Frederick, MD, USA) and housed at the College of Veterinary Medicine Division of Laboratory Animal Health (DLAH) Facility. Animals were maintained under constant conditions of light (12L:12D) and temperature (20–23.38 °C) with free access to pelleted food. Water was provided in glass water bottles ad libitum. The housing of animals in plastic cages and the use of glass bottles were designed to minimize background exposure to xenobiotics that may occur with resin-containing cages. The animal and euthanasia procedures were performed in accordance with the protocol (#2010-1742) approved by the Auburn University Institutional Animal Care and Use Committee (IACUC) and are based on recommendations from the Panel on Euthanasia of the American Veterinary Medical Association. A series of 35-day-old male rats were sacrificed by CO_2_ asphyxiation and their testes were collected. Leydig cells were isolated and cultured on collagen-coated 24-well plates, as described previously [15,22]. Only male rats were used in this study because the focus was on testicular Leydig cells, which are exclusively found in males.

**RIA Testosterone assay:** Testosterone (T) concentrations were assayed in aliquots of spent media using a tritium-based RIA validated for use with the rat-testosterone-specific antibody (Animal Reproduction and Biotechnology Laboratory, Colorado State University, Fort Collins, CO) [35]. The T-assay, using T standards containing 10–2000 pg/mL, has a sensitivity of 270 pg/mL and inter-assay and intra-assay coefficients of variation in the range of 3.38% to 9.56% and 5.69% to 9.84%, respectively [36]. Hormone production values are ng/10^6^ LCs.

**Culture of COS-7 and MA-10 cells:** COS-7 monkey kidney fibroblasts were obtained from the ATCC and grown in DMEM (Lonza) supplemented with 10% FBS (HyClone) [33,34]. MA-10 mouse LCs were kindly provided by Dr. Mario Ascoli (University of Iowa) [37]. MA-10 cells were grown in a manner similar to COS-7 cells. For RNA-Seq analysis, MA-10 cells were first grown in DMEM with 10% FBS, then maintained in phenol red-free DMEM supplemented with 5% charcoal-dextran treated serum for 24 h. Then, the cells were treated with either DMSO or 10 μM PCN for 24 h before harvesting the total RNA for RNA-Seq analysis.

**Transient transfection of COS-7 cells:** COS-7 cells were transiently transfected with pcDNA3, FLAG-pcDNA3, pcDNA3-hPXR, FLAG-pcDNA3-hPXR, or 3XFLAG-pcDNA3-hPXR plasmids using FuGENE 6 (Promega, Madison, WI, USA) [33,34]. After 48 h transfection, the cell lysates were collected using RIPA buffer for PXR protein expression analysis using the anti-mouse PXR antibody.

**RNA isolation:** RNA isolation, reverse transcription, and polymerase chain reaction were performed as previously described [32,38]. The total RNA was extracted from the rat primary LCs and MA-10 mouse LCs using the E.Z.N.A. Total RNA Kit (Omega Bio-Tek, Norcross, GA, USA). The quality and quantity of the total RNA was assessed using a NanoVuePlus Spectrophotometer (GE Healthcare).

**RNA-Seq analysis:** RNA-Seq analysis was performed as described previously [39]. The total RNA isolated from MA-10 cells was sent to Novogene Life Sciences Co., Ltd., Sacramento, CA, USA. for the PE150 RNA-seq service. Samples that passed the quality control (concentration > 25 ng/µL, RIN ≥ 7.5) were used for library preparation and subsequent RNA-seq. Unstranded paired-end read files were initially analyzed using the FASTQ error check feature of Chipster. Quality control was then conducted on the raw read files using FastQC. The trimmed reads were aligned to the mouse genome (mm10, GRCm38.95) using STAR (v. 2.5). Reads were mapped to each gene and counted using HTSeq (v. 0.6.1). The Maximal Mappable Prefix (MMP) method was employed to ensure precise mapping results for exon–exon junction reads. Differential expression analysis was performed using the DESeq2 in R version 3.6.2 with FDR cutoff α = 0.05.

**Western blotting analysis:** The total cell lysates of the rat primary LCs, MA-10, and CO-7 cells were collected in RIPA buffer containing a cocktail of protease inhibitors. Protein concentration was determined by a Bradford protein assay (Bio-Rad). Equal amounts of protein samples were resolved on an SDS-PAGE gel and then transferred onto a nitrocellulose membrane. The membranes were blocked, incubated with specific primary antibodies (Table 1), washed with Tris-buffered saline, and finally incubated with 1:1000 HRP-conjugated goat anti-rabbit (sc-2004) and donkey anti-goat (sc-2020) secondary antibodies (Santa Cruz Biotechnology, Dallas, TX, USA). The proteins were visualized using HyGLO Chemiluminescent HRP Antibody Detection Reagent (Denville Scientific, Holliston, MA, USA).

**Data and statistical analysis:** Data are shown as mean values ± SD. Analyses were performed using GraphPad Prism 9.0 (La Jolla, CA, USA). The statistical significance (*p* < 0.05) was evaluated by either a *t* test or one-way ANOVA, followed by a Tukey’s multiple comparisons test. For RNA-Seq analysis, differential expression analysis between control and PCN-treated groups was performed using DESeq2 in R version 4.3.2. Genes with a fold change greater than 1.5 and an adjusted *p*-value (padj) less than 0.05 were identified as differentially expressed genes (DEGs) [39].

## 3. Results

### 3.1. PXR Is Expressed in Rodent LCs

**Figure 1:**
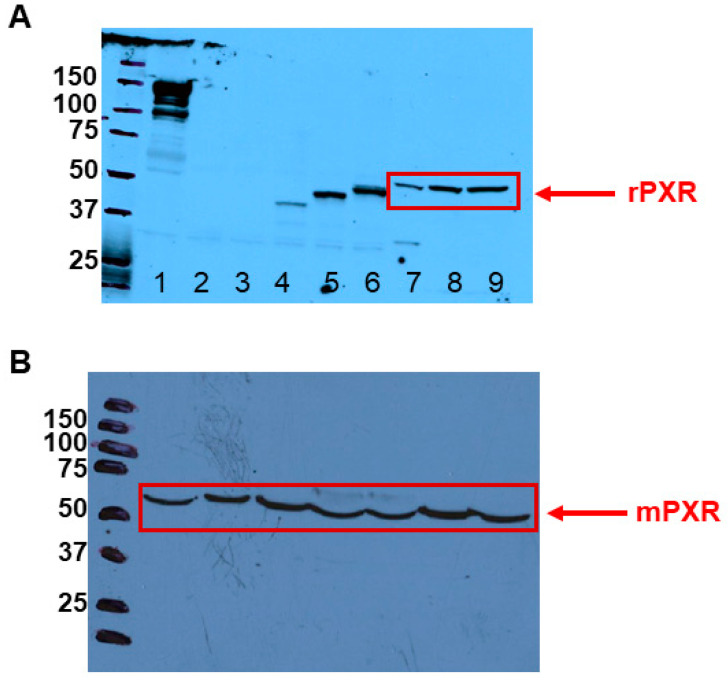
**PXR protein expression in rodent LCs.** (**A**). Protein expression of rPXR in rat primary LCs was determined by Western blotting analysis (*n* = 2). Image shown is from a representative experiment. Lane 1, dog liver; Lane 2, COS-7 cells transfected with pcDNA; Lane 3, COS-7 cells transfected with FLAG-pcDNA; Lane 4, COS-7 cells transfected with human PXR (hPXR); Lane 5, COS-7 cells transfected with FLAG-hPXR; Lane 6, COS-7 cells transfected with 3XFLAG-hPXR; and Lanes 7 to 9, rat primary LCs. Marker molecular weights represent KDa. (**B**). Protein expression of mPXR in mouse MA-10 cells was determined by Western blotting analysis (*n* = 2). Image shown is from a representative experiment. All Lanes (1 to 7), MA-10 cells. Marker molecular weights represent KDa.

We determined whether PXR is expressed in rodent LCs. RT-PCR and Western blot experiments revealed that rat PXR (rPXR) could be detected in rat primary LCs at both the transcript and protein levels (Figure 1A and Supplemental Figure S1A). Similarly, mRNA and protein expression were detected for mouse PXR (mPXR) in mouse MA-10 cells (Figure 1B and Supplemental Figure S1B).

Both rPXR and mPXR proteins were detected by the anti-mPXR antibody (Figure 1). The specificity of the PXR antibody was validated by probing overexpressed PXR, FLAG-PXR, and 3X-FLAG-PXR proteins in COS-7 cells (Figure 1). The specificity of rPXR and mPXR transcripts was validated and verified using negative RT and no-template controls, as well as by sequencing the corresponding molecular weight band (Supplemental Figure S1).

### 3.2. Pharmacological Activation of rPXR Regulates T Secretion by LCs

**Figure 2:**
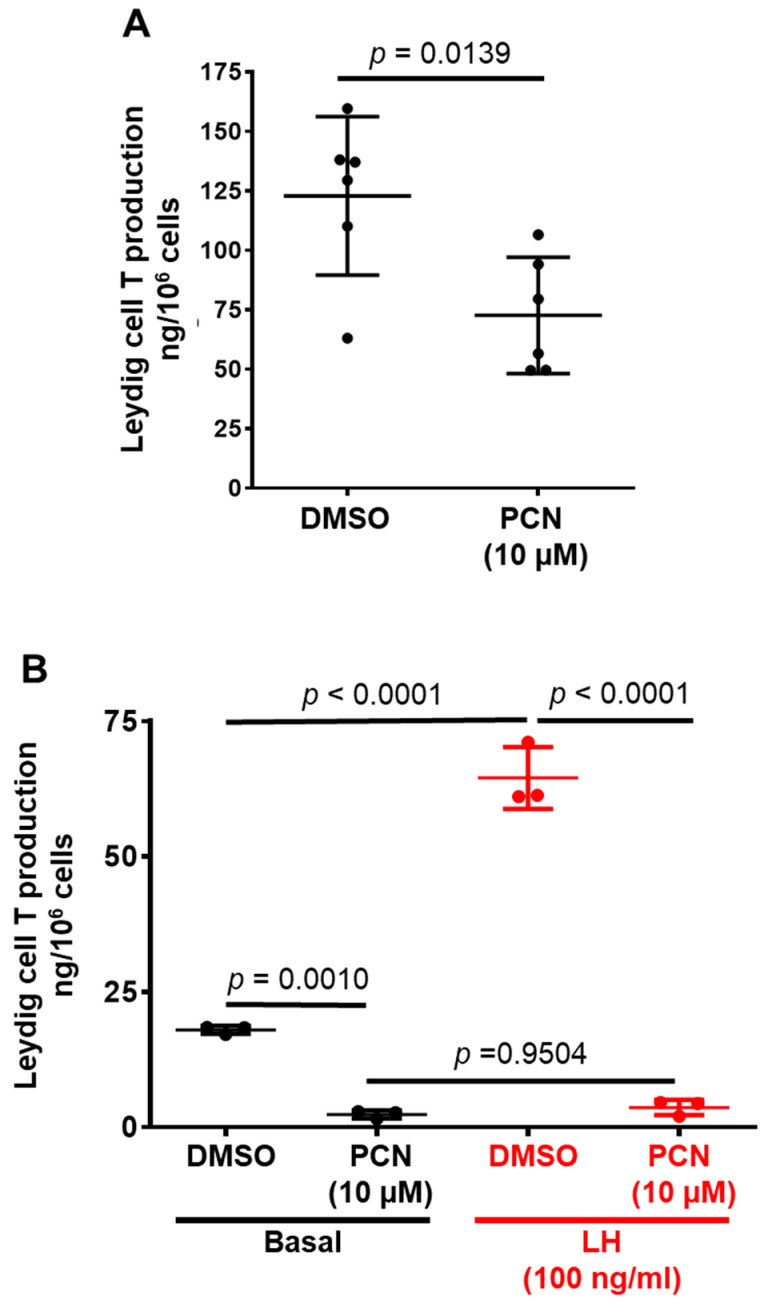
**Effect of pharmacological activation of rPXR on testosterone secretion by rat primary LCs.** (**A**). LCs were isolated from 35-day-old male rats. Pooled LCs were incubated in the culture media and treated with either DMSO (*n* = 6) or 10 µM PCN (*n* = 6) for 24 h. The media was aspirated and collected after 24 h treatments to measure the T levels using RIA. Data represent mean ± SD from six independent experiments. Statistical significance was determined using an unpaired Students *t* test. (**B**). Both DMSO and PCN-treated rat primary LCs were incubated in the culture medium without (basal) (*n* = 3) or with 100 ng/mL LH (LH) (*n* = 3) for 3 h. The media aliquots were collected to determine T production using RIA. Results are shown as the mean ± S.D. Determined by ANOVA and a Tukey’s multiple comparisons test.

Since rPXR is expressed in rat primary LCs, we asked the question of whether pharmacological activation of rPXR using the prototype rodent PXR agonist, PCN, affects T secretion. Indeed, compared to DMSO, PCN (10 µM) treatment for 24 h resulted in a significant decrease in T secretion by the LCs (*p* = 0.0139) (Figure 2A).

Luteinizing hormone (LH) is the primary regulator of T synthesis by LCs. We wanted to determine whether prior treatment with PCN affects LH-regulated T secretion by LCs. To evaluate this, we treated the LCs with DMSO or PCN (10 µM) for 24 h and then incubated the LCs with 100 ng/mL of LH for 3 h before measuring T secretion. As expected, LH significantly increased T secretion in DMSO-treated LCs (*p* < 0.0001). However, LH failed to increase T secretion in PCN-treated LCs (*p* = 0.9504) (Figure 2B). Together, these observations suggest that the pharmacological activation of rPXR leads to decreased T synthesis in rat primary LCs.

### 3.3. Pharmacological Activation of rPXR Affects Protein Expression of Key Steroidogenic Enzymes Involved in T Biosynthesis

**Figure 3:**
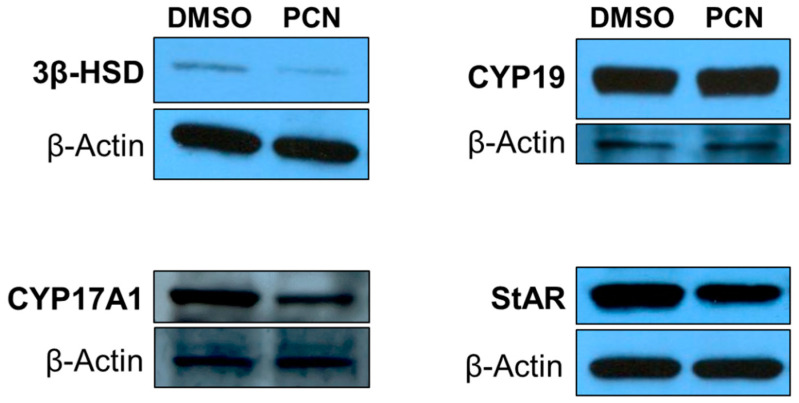
**Effect of pharmacological activation of rPXR on protein expression of key enzymes involved in testosterone biosynthesis in rat primary LCs**. Western blots (*n* = 2) showing the protein expression of 3β-HSD, CYP17A1, CYP19, StAR, and β-actin in rat primary LCs treated by DMSO or 10 µM PCN for 24 h. Data shown are from representative experiments.

We also determined protein expression of the main steroidogenic enzymes involved in T biosynthesis in the rat primary LCs treated with DMSO or PCN, as described in the previous section. Western blots showed that the main steroidogenic enzymes involved in T synthesis were down-regulated after PCN treatment (Figure 3). Notably, 3β-HSD and CYP17A1, which act successively to convert pregnenolone to T in LCs, were down-regulated by PCN treatment (Figure 3). We did not notice a considerable change between treatments in the expression of CYP19 (Figure 3). Together, these results suggest that the PCN-induced decrease in T secretion in rat primary LCs could in part be attributed to down-regulation of the steroidogenic enzymes involved in T biosynthesis.

### 3.4. PCN Induces Changes in Gene Expression of Key Players Involved in Steroidogenesis in Mouse MA-10 Cells

**Figure 4:**
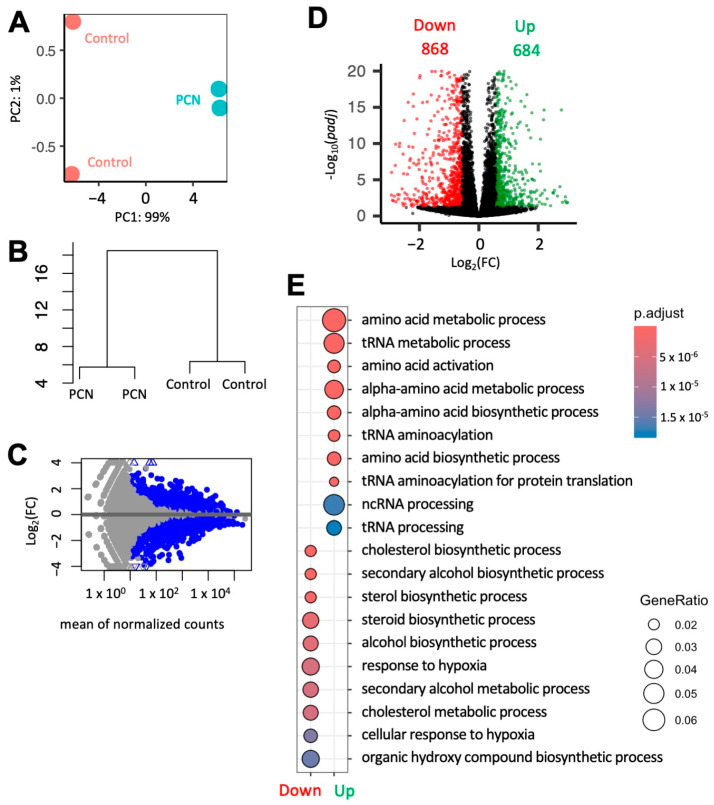
**RNA-seq analysis identifies 1552 DEGs in MA-10 cells.** (**A**) Principal component analysis and (**B**) dendrogram showing the clustering pattern of four RNA-seq samples. (**C**) MA plot and (**D**) volcano plot provide an overview of PCN (10 µM)-induced DEGs in an MA-10 cell transcriptome. (**E**) Gene ontology (GO) analysis shows top GO terms linked to up- and down-regulated DEGs.

**Figure 5:**
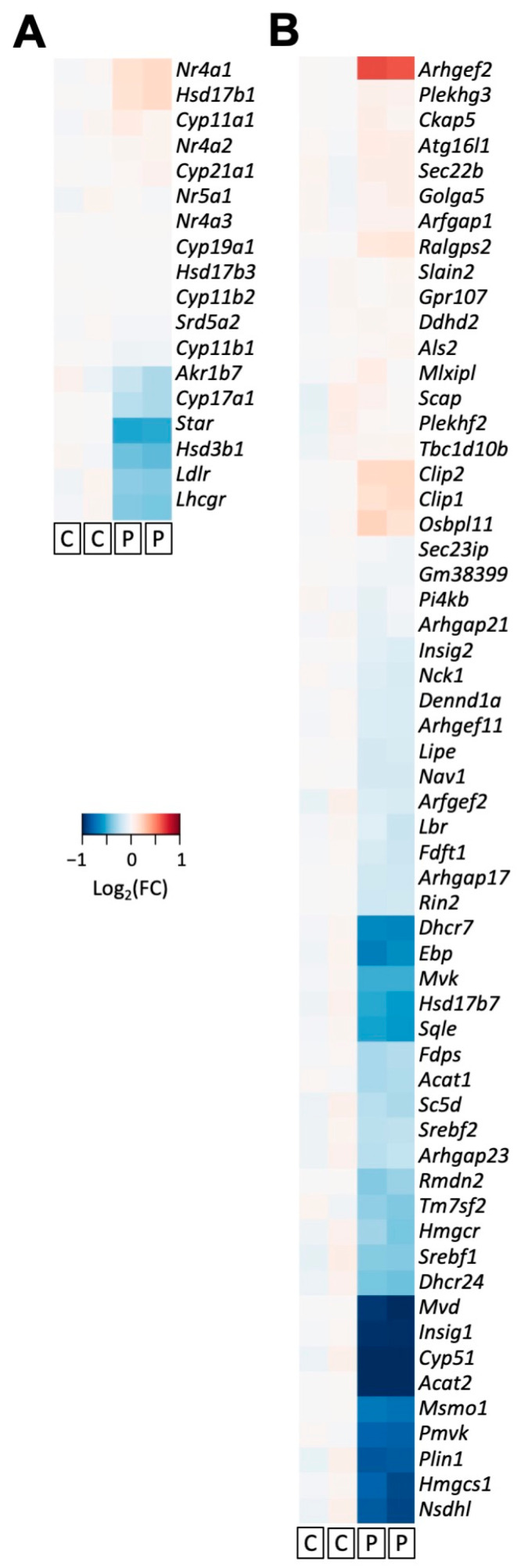
**Heatmaps of genes associated with (A) steroidogenesis and (B) cholesterol metabolism in MA-10 cells.** Heatmaps show diverse expression levels (log_2_ fold-change in FPKM value). To reduce the influence of low-expression genes, 10 was added to each FPKM value before calculating the fold change. C, Control (DMSO); P, PCN (10 µM).

Although MA-10 Leydig cells are not able to synthesize T due to the loss of 17β-HSD activity, these cells are an excellent system for studying the metabolism of other steroids, such as progesterone, and for gene expression analysis of steroidogenic pathways. In our RNA-Seq analysis from MA-10 cells, mPXR activation due to PCN resulted in significant changes at the transcriptomic level when compared to treatment with DMSO (Figure 4). Gene ontology analysis revealed that the genes down-regulated by PCN are associated with steroidogenesis and cholesterol metabolism (Figure 4E). PCN-induced down-regulation of the transcripts for 3β-HSD (encoded by *Hsd3b1*) and CYP17A1 (encoded by *Cyp17a1*) is consistent with the protein expression data from rat primary LCs (Figure 3 and Figure 5). Similarly, the lack of a PCN effect on the CYP19 (encoded by *Cyp19a1*) transcript in MA-10 cells was also in agreement with the protein expression in rat primary LCs (Figure 3 and Figure 5).

About 95% of T synthesis takes place in testicular LCs, and T is eventually derived from cholesterol in LCs. Steroidogenic acute regulatory protein (StAR) helps translocate cholesterol to mitochondria, and then cholesterol is metabolized to pregnenolone by the CYP11A1 enzyme at the inner mitochondrial membrane. Later, several steroidogenic enzymes in smooth endoplasmic reticulum enable conversion of pregnenolone to testosterone in LCs. Therefore, altered StAR and CYP11A expression could also lead to changes in T synthesis. While StAR appears to be marginally down-regulated at the protein level, it is significantly down-regulated at the transcript level (Figure 3 and Figure 5). Down-regulation of StAR after PCN treatment is consistent with the PCN-induced decrease in T secretion. PCN treatment had little effect on the transcript of *Cyp11a1* (Figure 5).

It is possible that the PCN-induced changes in the LCs are caused by PCN’s cytotoxicity. However, PCN did not induce any noticeable cytotoxicity in MA-10 cells even after 48 h treatment in cell viability assays or 72 h treatment in a cell morphology analysis (Supplemental Figures S2 and S3) [32,33,34,38,41].

## 4. Discussion

This is the first study to report that PXR is expressed in rat and mouse LCs and that the rodent PXR agonist, PCN, decreases T synthesis in rodent LCs by down-regulating the key steroidogenic enzymes involved in T biosynthesis. Our results are significant, as they provide a potential novel mechanism, within our current understanding, for the interplay between nuclear receptors and endocrine disruption in rodents.

The primary limitation of our study is that it falls short of conclusively demonstrating whether PCN regulates testosterone biosynthesis by LCs in a PXR-dependent manner. Therefore, future studies are warranted to determine whether PCN-induced changes in LCs are mediated through PXR by using PXR antagonist(s) as well as genetic approaches.

Two previous studies have reported that PXR is expressed in human testis but fall short of delineating whether PXR is expressed in LCs [42,43]. Our results show for the first time that PXR is expressed in the testicular LCs. Gray and Squires et al. have shown that rifampicin, the prototype agonist of human PXR, decreases sex steroid production by porcine primary LCs [44]. Our results show that activation of rPXR with the rodent PXR agonist PCN results in reduced T production in rat primary LCs. Both Gray and Squires et al. and our studies support the notion that pharmacological activation of PXR regulates T synthesis by LCs.

While some xenobiotics increase serum T levels, certain xenobiotics decrease serum T levels. Clinical drugs and supplements, such as phenobarbital, dexamethasone, licorice, and genistein, decrease T levels [5,6,9,11,12,13]. Similarly, environmental chemicals, such as PCB, BPA, ethinylestradiol, PFOS, PFOA, triclosan, cypermethrin, and methoxychlor, also decrease T levels [14,15,16,17,18,19,20,21,22,45]. Homeostasis of T could be affected by changes in testicular synthesis and/or hepatic metabolism. Therefore, reduced T levels could be because of decreased synthesis, increased metabolism, or a combination of both. As these xenobiotics have the potential to activate PXR, it is possible that they act through PXR in LCs to decrease T levels by impairing T synthesis. Based on our results, it is worthwhile to investigate PXR wild-type, PXR knockout (whole body or conditional), and PXR humanized (whole body or conditional) mouse models to determine whether these xenobiotics decrease T synthesis by LCs in a PXR-dependent manner. Incorporating humanized PXR models will allows us to determine the species dependency, as PXR is known to be activated by ligands in a species-specific manner.

In conclusion, our results suggest that pharmacologic activation of PXR can lead to decreased T biosynthesis in rodent LCs by down-regulating the expression of the steroidogenic enzymes involved in T biosynthesis.

## Figures and Tables

**Table 1:** Primary antibodies used in Western blot analysis.

Target Protein	Dilution	Host Species	Catalog No/Manufacturer or Reference
PXR	3000	Rabbit polyclonal	Provided by Dr. Rakesh Tyagi [40]
β-actin	2000	Goat polyclonal	sc-1616, Santa Cruz Biotechnologies
CYP17A1	20,000	Goat polyclonal	sc-46081, Santa Cruz Biotechnologies
3β-HSD	2000	Rabbit polyclonal	sc-28206, Santa Cruz Biotechnologies
CYP19	500	Rabbit polyclonal	sc-30086, Santa Cruz Biotechnologies
StAR	5000	Rabbit polyclonal	sc-25806, Santa Cruz Biotechnologies

## Data Availability

The data presented in this study are available on request from the corresponding authors.

## Supplementary Materials

The following supporting information can be downloaded at: https://www.mdpi.com/article/10.3390/jox14030071/s1, Figure S1: PXR mRNA expression in rodent LCs; Figure S2: Effect of PCN on viability of MA-10 cells; Figure S3: Effect of PCN on morphology of MA-10 cells.
