# Supplementary material for: Pregnenolone 16-Alpha Carbonitrile, an Agonist of Rodent Pregnane X Receptor, Regulates Testosterone Biosynthesis in Rodent Leydig Cells
Source: J Xenobiot. 2024 Sep 16;14(3):1256–67. doi: 10.3390/jox14030071 (PMC11417858; doi:10.3390/jox14030071)
Supplement: Supplementary file 1 [file jox-14-00071-s001.zip › jox-3141725-supplementary.pdf]

Supplemental Methods

RT-PCR analysis: Reverse transcription was performed with the iScript cDNA Synthesis Kit (Bio-Rad). Polymerase chain reaction was performed for 40 cycles by using the PerfeCTa SYBR Green FastMix (Quanta BioSciences) and CFX96 Touch Real-Time PCR Detection System (Bio-Rad). Transcripts of the rPXR and mPXR were amplified using the gene-specific primers **(Supplemental Table1)**. PCR products were run on 2% agarose gels and detected by ethidium bromide. Purified PCR products were sequenced to verify the identity of the RT- PCR products.

Supplemental Table 1: Forward (F) and reverse (R) primers used for RT-PCR of rPXR and mPXR.

**Gene/Primer sequence**	**Gene Bank Accession no**
rPXR<br>F: 5′-TGATCATGTCTGATGCCGCTG-3′<br>R: 5′-GAGGTTGGTAGTTCCAGATGCTG-3′	NM_052980
mPXR<br>F: 5′-GGGATAGGGTTACAGCACGA-3′<br>R: 5′-CCACCGCCATAGTTCTCATC-3′	NM_010936

Cell viability assays: Cell viability assays were performed in MA-10 cells. The cells were treated with DMSO or 10 µM PCN for 48 h before measuring cell viability using CellTiter-Glo Luminescent Cell Viability Assays (Promega) [1-5].

Cell morphology analysis: Cell morphology of MA-10 cells was visualized after treating the cells DMSO or 10 µM PCN for 72 h using a Revolve 4 microscope (ECHO).

Supplemental Results

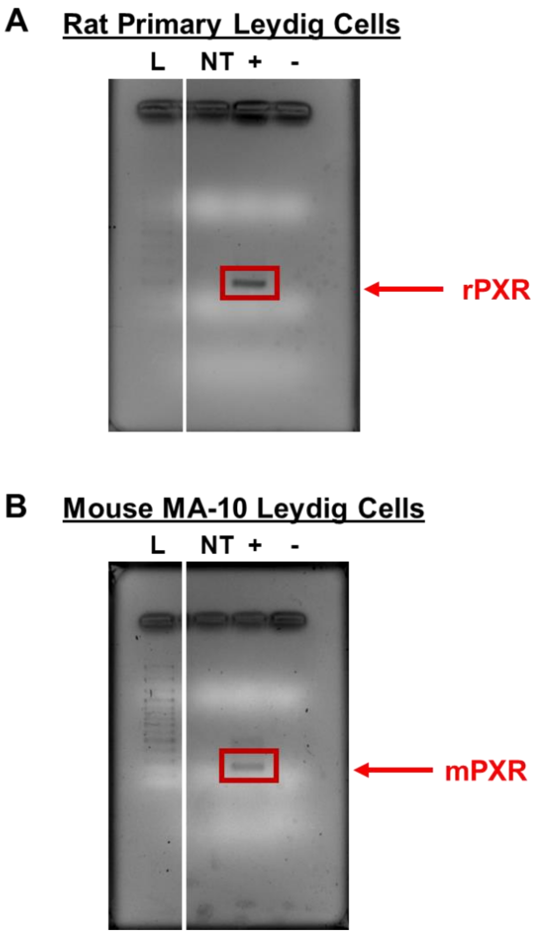

Supplemental Figure 1. PXR mRNA expression in rodent LCs. Determination of the transcript for rPXR in rat primary LCs (A) and mPXR in mouse MA-10 cells (B). RT-PCR products were run on a 2% gel. Bands were observed at the expected size in reverse transcription (+), but no signal was observed in negative reverse transcription (-) control and no-template (NT) control. Identity of the band was verified by sequence analysis. L, 100-bp ladder.

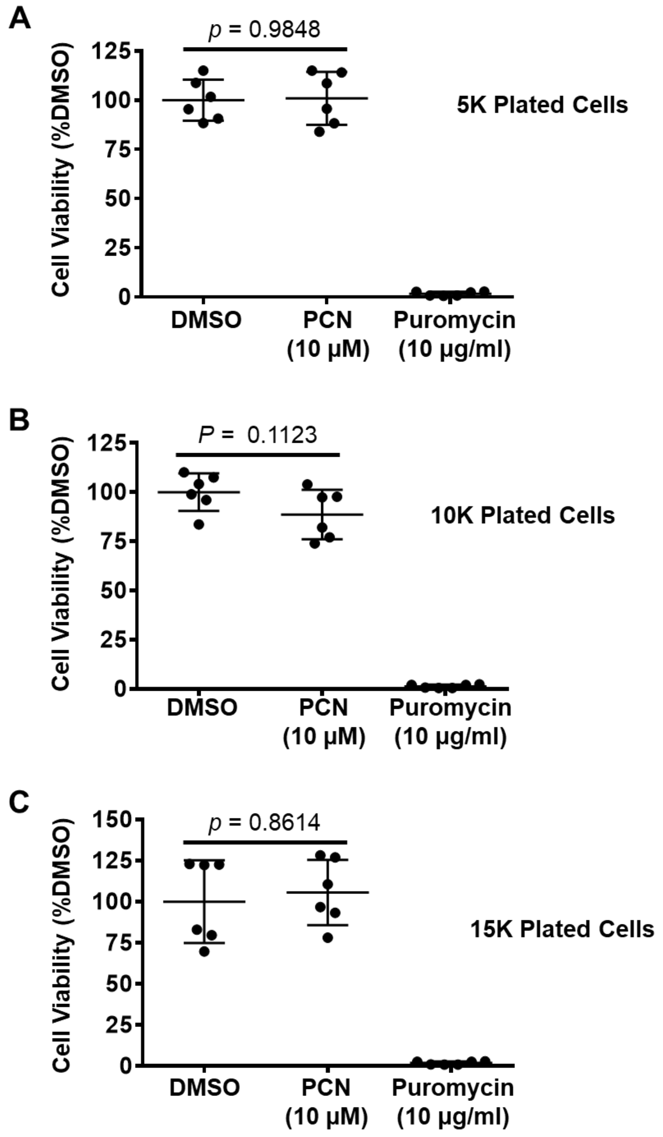

Supplemental Figure 2. **Effect of PCN on viability of MA-10 cells.** MA 10 cells were seeded at 5K (A), 10K (B), and 15K (C) densities and incubated overnight. The cells were then treated with DMSO or 10 µM PCN for 48 h before measuring viability by using CellTiter-Glo reagent. Puromycin was used as a positive control for cytotoxicity. Viability of DMSO-treated cells was expressed as 100%. Results are shown as mean ± SD. * $p < 0.05$; compared with DMSO by ANOVA and Tukey's multiple comparisons test.

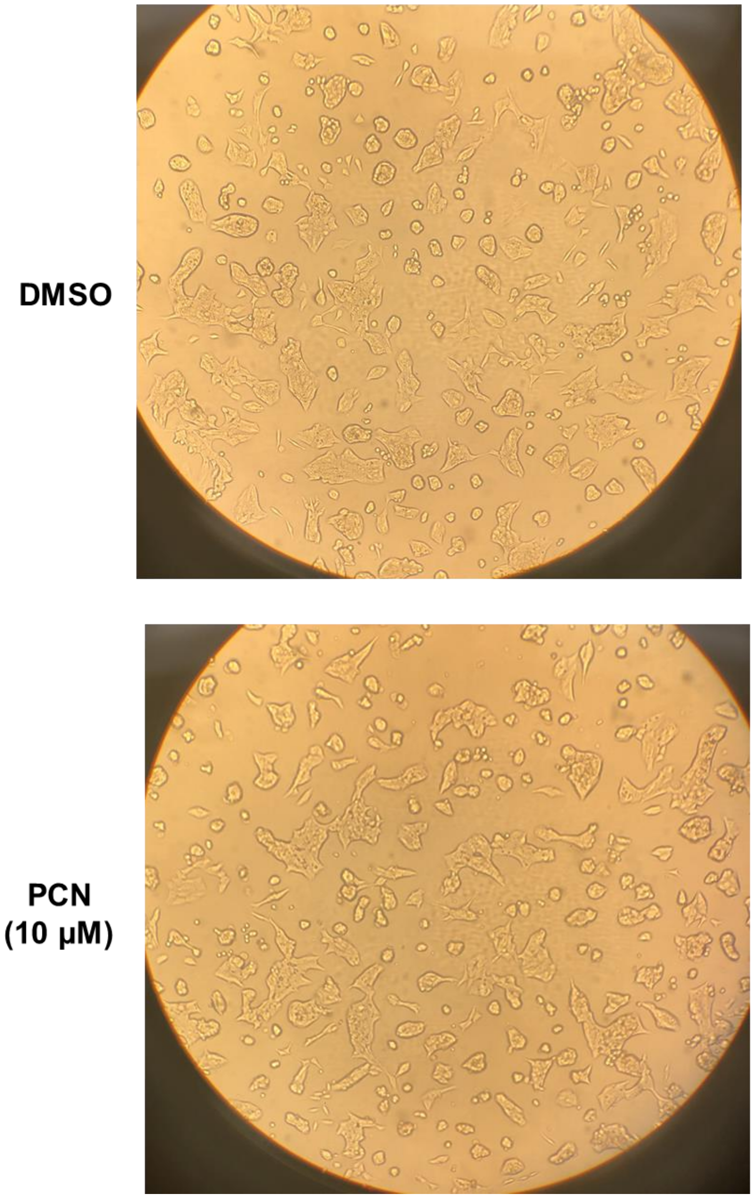

Supplemental Figure 3. Effect of PCN on morphology of MA-10 cells. MA 10 cells were seeded and incubated overnight. The cells were then treated with DMSO or 10 µM PCN for 72 h before taking images using a Revolve 4 microscope (ECHO). The representative images are shown.

References

1. Pondugula, S.R.; Ferniany, G.; Ashraf, F.; Abbott, K.L.; Smith, B.F.; Coleman, E.S.; Mansour, M.; Bird, R.C.; Smith, A.N.; Karthikeyan, C.; et al. Stearidonic acid, a plant-based dietary fatty acid, enhances the chemosensitivity of canine lymphoid tumor cells. *Biochemical and biophysical research communications* **2015**, *460*, 1002-1007, doi:10.1016/j.bbrc.2015.03.141.
2. Pondugula, S.R.; Flannery, P.C.; Abbott, K.L.; Coleman, E.S.; Mani, S.; Temesgen, S.; Xie, W. Diindolylmethane, a naturally occurring compound, induces CYP3A4 and MDR1 gene expression by activating human PXR. *Toxicology letters* **2015**, *232*, 580-589, doi:10.1016/j.toxlet.2014.12.015.
3. Pondugula, S.R.; Flannery, P.C.; Apte, U.; Babu, J.R.; Geetha, T.; Rege, S.D.; Chen, T.; Abbott, K.L. Mg2+/Mn2+-Dependent Phosphatase 1A Is Involved in Regulating Pregnane X Receptor-Mediated Cytochrome p450 3A4 Gene Expression. *Drug Metab Dispos* **2015**, *43*, 385-391.
4. Abbott, K.L.; Chaudhury, C.S.; Chandran, A.; Vishveshwara, S.; Dvorak, Z.; Jiskrova, E.; Poulikova, K.; Vyhlidalova, B.; Mani, S.; Pondugula, S.R. Belinostat, at Its Clinically Relevant Concentrations, Inhibits Rifampicin-Induced CYP3A4 and MDR1 Gene Expression. *Mol Pharmacol* **2019**, *95*, 324-334, doi:10.1124/mol.118.114587.
5. Flannery, P.C.; Abbott, K.L.; Pondugula, S.R. Correlation of PPM1A Downregulation with CYP3A4 Repression in the Tumor Liver Tissue of Hepatocellular Carcinoma Patients. *Eur J Drug Metab Pharmacokinet* **2020**, *45*, 297-304, doi:10.1007/s13318-019-00595-3.
